# 

**Published:** 1897-09

**Authors:** 


**Mississippi Valley Medical Association.** — Arrangements are now about completed for the meeting of the Association at Louisville on October 3, 6, 7, 8, 1897. The different passenger associations have granted a round-trip rate of one and one-third fare on the certificate plan. The sessions will be held at the Liederkranz Hall, and the headquarters will be at the Louisville Hotel.

CONGRESS OF THE NATIONAL PRISON ASSOCIATION AT AUSTIN. The 26th Annual Congress of the National Prison Association of the United States, will be held in Austin, Texas, October 16th to 20th prox. Gen. Rocliff Brinkerhoff, of Mansfield, O., is President. Among the honorary vice-presidents we notice the name of Hon. Wm. Clements, of New Braunfels, Texas. Among the directors is Hon. T. J. Goree, of Galveston. Gov. Hogg is one the Committee of Criminal Law Reform. As announced in our July issue, a Committee of Reception, composed of prominent business men of Austin, have made the neccessary arrangements for the meeting; and a general invitation to attends and participate in discussions is extended to—"not only prison officers, but also to legislators, judges, educators, clergymen and all others who are interested in the prison question, and have given special study to the solution of its problems," says the President in his address ("Doctors" are not mentioned, specifically, but doubtless it was intended to include them in the last paragraph.)

Few of the lay people have any idea of the importance of this meeting or of the objects of this Association. That our readers may appreciate both, we quote from the address of the President delivered at the last meeting, Milwaukee, Wis., September, 1896, the following:

"The importance of the prison question cannot well be overestimated, for in its solution the very existence of free institutions is at stake. If safety to persons and property cannot be maintained, a stronger form of government becomes a necessity.

"In this question every citizen ought to be actively interested, for unless protected, he is at the mercy of the criminal. To secure this protection an army of policemen larger than the army of the United States is constantly on guard, and must be paid. In addition, all the machinery of the criminal courts must be maintained; penitentiaries for an avergage of one hundred thousand convicts must be supported, and in every county there is a jail, so that the money expenditures alone counts into the millions every year. * * *

"In the study of this problem, the National Prison Association has been at work for a quarter of a century. * * * Primarily the objects of this Association was to bring together the men and women actually engaged in the administration of prisons and reformations, with a view to an exchange of experiences, and the consideration of methods: and that, still, is an important part of its work; but the Congress has long since enlarged its boundaries so as to include the whole range of subjects which enter into the suppression or prevention of crime, whether punitive, legislative, educational or otherwise." * * *

When it is recalled that under existing systems crime increased in the United States in the four decade from 1850 to 1890, at a ratio nearly three times greater than the increase of population; that is, while population increased 170 per cent. the criminals increased 445 per cent. (See paper by Dr. Daniel "Reform in Criminal Jurisdiction," in *Transactions*, *Texas State Medical Association*, *Journal of the American Medical Association*, TEXAS MEDICAL JOURNAL, etc.), it is evident that there is something radically wrong with that system, and that a change is imperatively demanded. Our penal system makes criminals.

It is to be hoped that this Congress will recommend some legislation looking to the *prevention of criminals*, as well as to the prevention of crime. Hanging does not prevent crime; but rather, it seems to us to suggest it, or excite to it. Why then, hang a criminal? Let us try emasculation and solitary confinement for the incorrigibles.

---

THE *American Journal of Surgery and Gynecology*, in announcing the election of Prof. Jeff. Davis Griffith, M. D., of Kansas City, to the presidency of the National Association of Military Surgeons, says that Dr. Griffith is a nephew of Jefferson Davis, the President of the late Southern Confederacy.

Brother Lanphear is away off here. We have known Jeff Griffith since he was a baby. He is no kin to his illustrious namesake, and Lanphear was misled, doubtless, by the name. We know whereof we speak. Jeff Davis was colonel of the First Mississippi Rifles, an infantry regiment, and won his military fame at Buena Vista, during the Mexican war. Major, later Colonel, then General Richard Griffith, of Mississippi, Dr. Griffith's father, was his adjutant, and a warm friendship existed between the adjutant and colonel, which endured to the end. Dr. Griffith's mother was a Whitfield, of the Alabama stock of Whitfield's.

Prof. Griffith is Surgeon General of the Missouri militia, and Lanphear says he is the first president of the Association ever elected outside the U. S. army. If the doctor is not a kinsman of the illustrious Jeff Davis, he is none the less an honorable scion of an honored sire, and is a soldier by heredity. General Griffith lived to serve with brilliant distinction in the Confederate army, and fell, we believe, in one of the battles around Richmond. We congratulate "Jeff" on this recognition of his soldierly qualities, and his many social and professional graces.

